# Muscular Development in *Urechis unicinctus* (Echiura, Annelida)

**DOI:** 10.3390/ijms21072306

**Published:** 2020-03-26

**Authors:** Yong-Hee Han, Kyoung-Bin Ryu, Brenda I. Medina Jiménez, Jung Kim, Hae-Youn Lee, Sung-Jin Cho

**Affiliations:** 1 School of Biological Sciences, College of Natural Sciences, Chungbuk National University, Cheongju, Chungbuk 28644, Korea; yhhanbio@naver.com (Y.-H.H.); lightbean1126@gmail.com (K.-B.R.); brenda.medina@geo.uu.se (B.I.M.J.); 2 Department of Earth Sciences, Paleobiology, Uppsala University, Villavägen 16, 75236 Uppsala, Sweden; 3 Department of Molecular and Cell Biology, University of California, 539 LSA, Berkeley, CA 94720-3200, USA; jkim81@berkeley.edu

**Keywords:** Echiura, *Urechis unicinctus*, musculature, striated muscle, smooth muscle

## Abstract

Echiura is one of the most intriguing major subgroups of phylum Annelida because, unlike most other annelids, echiuran adults lack metameric body segmentation. *Urechis unicinctus* lives in U-shape burrows of soft sediments. Little is known about the molecular mechanisms underlying the development of *U. unicinctus*. Herein, we overviewed the developmental process from zygote to juvenile *U. unicinctus* using immunohistochemistry and F-actin staining for the nervous and muscular systems, respectively. Through F-actin staining, we found that muscle fibers began to form in the trochophore phase and that muscles for feeding were produced first. Subsequently, in the segmentation larval stage, the transversal muscle was formed in the shape of a ring in an anterior-to-posterior direction with segment formation, as well as a ventromedian muscle for the formation of a ventral nerve cord. After that, many muscle fibers were produced along the entire body and formed the worm-shaped larva. Finally, we investigated the spatiotemporal expression of *Uun_st-mhc*, *Uun_troponin I*, *Uun_calponin*, and *Uun_twist* genes found in *U. unicinctus*. During embryonic development, the striated and smooth muscle genes were co-expressed in the same region. However, the adult body wall muscles showed differential gene expression of each muscle layer. The results of this study will provide the basis for the understanding of muscle differentiation in Echiura.

## 1. Introduction

Echiurans (also called spoon worms) are a morphologically unique group of annelids that include approximately 165 species [1], most of which lack segmentation as adults, although they do possess annelid-like morphological and developmental features, including the organization of a larval nervous system [2]. Traditionally, echiurans have been excluded from annelids and considered a separate phylum because of their unsegmented body, and were regarded as close relatives of annelids based on developmental and morphological characteristics [3,4,5]. However, recent morphological and molecular data support the classification of echiurans as a polychaete group within Annelida [6,7,8]. These new findings demand further studies to help resolve the phylogenetic position of Annelida. *Urechis unicinctus* is an echiuran species that inhabits burrows in soft sediments in intertidal areas [4,5,9]. The *Urechis* genus may hold important clues to the genetic basis of evolutionary gain and loss of segmentation, due to its nested position within the Annelida (i.e., sister to capitellid polychaetes) [10,11]. Recently, the transcriptome analysis during the development of *U. unicinctus* has been reported [12,13]. However, little is known about the molecular basis of muscular development in *U*. *unicinctus*. 

Among the echiuran species, until now, neurogenesis from the early stage to the metameric stage has been studied in *Urechis caupo* embryos [3], but the muscle differentiation from the early trochophore stage to the adult stages in echiuran species has not been reported. The musculature of the Capitellida is composed of a closed outer layer of circular fibers similar to that of clitellate oligochaetes, even though ring muscles are absent in various polychaete clades [14]. In contrast to the uniform muscle layers found in clitellates, polychaetes exhibit a diversity of complex muscle patterns of longitudinal muscle bands without distinct circular muscles along the body axis [14,15]. In sipunculans, the body wall musculature consists of not only numerous longitudinal muscles around retractors but also homogeneously arranged circular muscles along the entire anterior–posterior axis. With the comparative studies of muscle system in annelids [16], *U. unicinctus* can also help to understand the ancestry of the muscular systems in annelids and lophotrochozoan as a whole. 

In addition, previous studies have shown that the expression of striated and smooth muscle genes in polychaete belonging to Annelida is similar to that of vertebrates [17]. However, the striated and smooth muscles of *U. unicinctus*, one of the phylogenetically related but morphologically different forms of polychaete, have not been studied yet. The striated muscle myosin heavy chain is expressed in all myocytes and duplicated into cardiac, fast skeletal, and slow skeletal isoforms in vertebrates [18]. Troponin I, one of the heart and skeletal muscle protein troponin complexes, has been used as a striated muscle marker [19,20]. Among smooth muscle markers, calcium-binding proteins, such as calponin [21], have been used as marker genes, and twist has been used as a mesoderm marker, which are genetically conserved in bilateria [22].

Here, we investigated the development of *U. unicinctus* in embryos, larvae, metameric transitional stages, and juvenile worms. To better understand the timing and development of the mesoderm and muscles in this species, we utilized a combination of immunohistochemistry and in situ hybridization. We also performed experiments to examine the expression patterns of genes that may be involved in the process of muscle differentiation within Echiura.

## 2. Results and Discussion

### 2.1. Embryonic Development of U. unicinctus

We investigated the general development of *Urechis unicintus* from zygote to larval stages using immunohistochemistry because few studies until now have confirmed the spatial orientation of microtubules during the cleavage stage and the cilial differentiation from trochophore to juvenile stages in a series. 

**Figure 1:**
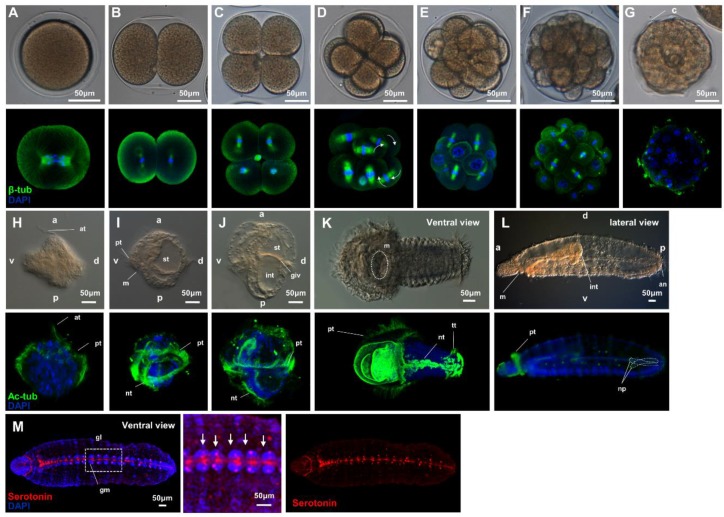
Confocal images of beta-tubulin and acetyl-tubulin staining in *U. unicinctus* during developmental stages. The β-tubulin-labeled microtubule filament appears in green, DAPI (4’,6-diamidino-2-phenylindole)-stained DNA is in blue. (**A**–**G**) Cleavage stages: (**A**) zygote, (**B**) 2 cells, (**C**) 4 cells, (**D**) 8 cells, (**E**) 16 cells, (**F**) 32 cells, and (**G**) blastula. The acetylated-tubulin-labeled cilia are shown in green, DAPI-labeled DNA is shown in blue. (**H**–**J**) Trochophore stages: (**H**) early trochophore, (**I**) mid-trochophore, (**J**) late-trochophore, (**K**) early-segmentation stage, and (**L**,**M**) worm-shaped larva. The arrowhead is nephridia. Serotonin is shown in red. Abbreviations: c, cilia; at, apical tuft; pt, protroch; nt, neurotroch; tt, telotroch st, stomach; int, intestine; glv, gastro-intestinal valve; ls, larval stomach; an, anus; m, mouth; np, nephridia; gl, ganglion. Bright-field images (**A**–**G**) were taken with a Nikon SMZ18 microscope; bright-field image (**H**–**L**) were taken with a Leica DM6 B microscope. All immunohistochemical images were taken with an LSM 710 confocal microscope (Carl Zeiss). Scale bars are 50 μm in length.

To identify the cleavage pattern of *U. unicinctus* after fertilization and the cilial differentiation from trochophore to juvenile stages, we performed immunohistochemistry and confocal microscopy with antibodies recognizing beta-tubulin and acetylated alpha-tubulin. The first two cleavages were equal and almost perpendicular (Figure 1B,C), as in other equal-cleaving embryos. From the third cleavage, the beta-tubulin antibody showed the tilt of microtubules in a dextral manner (white arrow), which is a typical pattern for spiralian development (Figure 1D–F). Cilia were observed on the surface of the embryo, and the rotation of the embryo was recognized (Figure 1G). In summary, embryos of the *U. unicinctus* displayed a holoblastic cleavage up to the 8-cell stage (Figure 1A–D) and the typical spiral cleavage pattern until the blastula stage (Figure 1E–G) seen in annelids.

The larva of *U. unicinctus* exhibited free-swimming ability from the trochophore stage, approximately 24 hours after fertilization (Figure 1H). During the early trochophore stage, an apical tuft appeared at the episphere of the larva, and a prototroch was observed (Figure 1H). In the mid-trochophore stage (between the 2nd and 4th day post-fertilization; dpf), the neurotroch appeared in the ventral region, and the mouth and stomach were gradually formed for feeding, which begins between the 2nd and 3rd dpf (Figure 1I). The stomach and intestine were distinctly divided (Figure 1J), while the gastro-intestinal valve membrane was observed in late trochophores (Figure 1J). In the early segmentation larval stage, segmentation was clearly observed and the neurotroch was elongated along an anterior-to-posterior direction (Figure 1K). The neurotroch was extended along the anterior-posterior axis, and a telotroch was observed on the posterior side (Figure 1K). Finally, in the worm-shaped larval stage, the body was elongated and tube-shaped, the cilia were lost, and the long intestine was connected by a single tube as well as a pair of nephridia on the posterior end (Figure 1L). Ganglionic masses were found in the ventral region and paired peripheral ganglia formed along the ventral nerve cord (Figure 1M). Serotonin immune-reactive cells were present in the ganglia of each segment, a trait that is typical of Annelida, comprised of repetitive units of neurons containing particular neurotransmitters (Figure 1M). In the worm-shaped larva stage, the worm was able to burrow in the mud. Most specimens measured about 1 mm in length, which might extend to almost 2 mm (Figure 1L). 

### 2.2. Muscle Differentiation 

**Figure 2:**
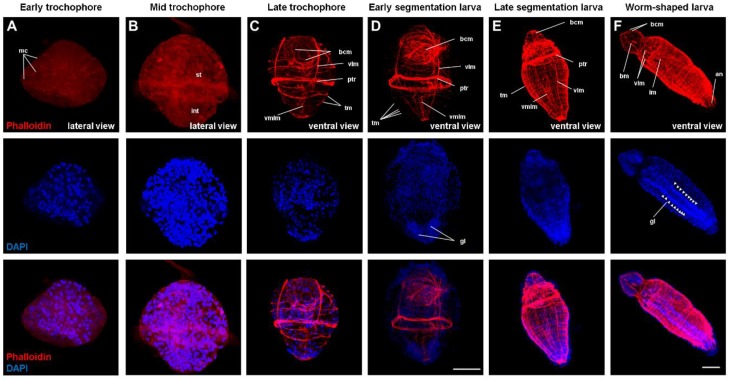
Confocal images of phalloidin and DAPI staining in *Urechis unicinctus* developmental stages. (**A**) Early trochophore, (**B**) mid-trochophore, (**C**) late trochophore, (**D**) early-segmented larva, (**E**) late-segmented larva, and (**F**) worm-shaped larva. Abbreviations: an, anus; bm, buccal muscle; bcm, buccal circular musculature; im, intestinal muscle; mc, myocyte; ptr, prototroch muscle ring; vmlm, ventromedian longitudinal muscle; st, stomach; int, intestine; tm, transverse muscle; vlm, ventrolateral longitudinal muscle; vnc, ventral nerve cord. Images **A**–**F** were taken with an LSM 710 confocal microscope, scale bar 100 μm. Images **A**–**D** are the same magnification.

To elucidate the differentiation of muscles from the early trochophore to juvenile stages, we performed overall phalloidin staining. Myocytes became apparent at the early trochophore stage (Figure 2A). Myocytes spread throughout the whole body, and the separation of stomach and intestine was apparent at the mid-trochophore stage (Figure 2B). In the late trochophore stage, a bundle of muscle fibers was clearly formed (Figure 2C). Even though several circular muscles were added in an anteroposterior gradient during this stage, they were incomplete in the earlier developmental stages (Figure 2A,B), extending from the ventral toward the dorsal side, where they were finally fused during the late trochophore stage. There was a tendency for increased circular musculature during the early segmented larval stage. At this stage, the ventral nerve cord was observed under nuclear staining with DAPI (4’,6-diamidino-2-phenylindole) (Figure 2D). The transverse muscles formed an almost continuous sheath, an intestinal muscle was observed in the worm-shaped larva, and the ganglia of the ventral nervous system were apparent at the same stage (Figure 2F). Transverse muscles increased throughout the body and formed a muscular mesh (Figure 2D–F). In the late trochophore, buccal muscles were formed for feeding, and the buccal muscle fibers increased in the center of the mouth for smooth peristalsis. As the development proceeded, these buccal muscles moved toward the anterior direction, along with the prototroch muscle ring (Figure 2D–F). The prototroch ring muscle directly below the prototroch was a solid band of muscle fibers that is essential for trochophore swimming using cilia. For similar reasons, the ventromedian longitudinal muscle and ventrolateral longitudinal muscle are structurally important for flexible movement, so the ventromedian longitudinal muscle is located at the center, and several ventrolateral longitudinal muscles are located on each side for support (Figure 2C–F). Unlike polychaete, which has distinct longitudinal muscles and weakly developed transverse muscles (circular muscles) or even absent [14,15], it was shown that the longitudinal and transverse muscles of *Urechis unicinctus* were developed simultaneously from anterior to posterior body axis. This may be correlated with the lifestyle of *U. unicinctus* living in U-shape burrows of soft sediments, whereas polychaeta taxa use their parapodia for walking and swimming, thus selecting for longitudinal musculature rather than circular muscle. These results provide information on the differentiation of the overall musculature according to lifestyle, as well as fundamental findings for the study of *U. unicinctus*, an Annelida species. 

### 2.3. Expression of Muscle-Related Genes During U. unicinctus Development

**Figure 3:**
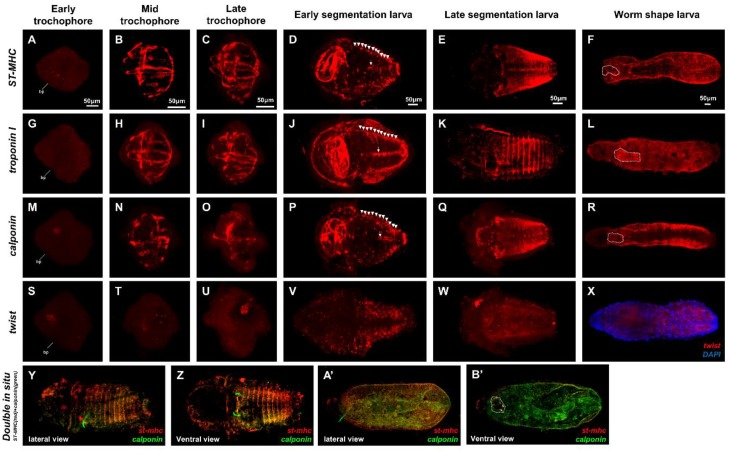
Expression patterns of *Uun_st-mhc*, *Uun_troponin I*, *Uun_calponin*, and *Uun_twist* genes in *U. unicinctus*. Whole-mount in situ hybridization (ISH) of *U. unicinctus* embryos showing the expression pattern of *Uun_st-mhc* (**A**–**F**), *Uun_troponin I* (**G**–**L**), *Uun_calponin* (**M**–**R**), and *Uun_twist* (**S**–**X**). Double ISH (*Uun_st-mhc and Uun_calponin*) (**Y**–**B’**). All images are oriented anteriorly to the left. Panels (**A**–**C**), (**G**–**I**), (**M**–**O**), (**S**–**U**), and (**Y**,**A’**) represent lateral views. Panels (**D**–**F**), (**J**–**L**), (**P**–**R**), (**V**–**X**), and (**Z**,**B’**) are ventral views. See the text for detailed descriptions of the expression patterns. (**A**–**F**) *Uun_st-mhc*, (**G**–**L**) *Uun_troponin I*, and (**M**–**R**) *Uun_calponin* expression throughout the esophagus, anterior to the posterior body muscle in a mid-late trochophore. In the early segmentation larval stage, specific expression was observed in the transversal muscles (white arrowhead) and ventromedian longitudinal muscles (white arrow) in the segment, whereas in the late metameric stage, the expression was strong in the transverse muscles and there was no expression in the buccal muscles. Specific expression was observed in the foregut (dotted white line) in the juvenile stage (**F**,**L**,**R**). *Uun_twist* is expressed in the mesoderm lineage region in the trochophore stage (**S**,**T**,**U**), in the segmented region in the segmentation larva stage (**V**,**W**), and in the worm-shaped larva (**X**) between the body muscle (DAPI) and internal organs. Co-expression of *Uun_calponin* and *Uun_st-mhc* in early segment larva and worm-shaped larva. Two-color fluorescent ISHs for *Uun_calponin* (green), *Uun_st-mhc* (red), and merged views (**Y**,**Z**,**A’**,**B’**). Abbreviations: bp, blastpore. Scale bars are 50 μm in length.

We identified three muscle-related genes: *striated muscle myosin heavy chain*, *calponin* (a smooth muscle calcium transducer), and *troponin I* (a calcium transducer for striated muscle). To identify the early mesoderm, we analyzed *twist* gene expression (Figure 3).

*Uun_st-mhc*, *Uun_troponin I*, *Uun_calponin*, and *twist* were expressed in a mesoblast cell and the blastpore region at the early trochophore stage (Figure 3A,G,M,S). 

*Uun_st-mhc*, *Uun_troponin I*, and *Uun_calponin* were strongly expressed in the pharyngeal muscles and the longitudinal and circular muscles in mid and late trochophores, the stages at which muscle fibers form (Figure 3C,I,O). At this time, these muscles began to excrete digested food (Figure 3B,C,H,I,N,O). The *twist* gene was expressed in the mesodermal band region during the early to late trochophore stages (Figure 3S,T,U). *Uun_st-mhc*, *Uun_troponin I*, and *Uun_calponin* genes were strongly expressed, not only in the ventral transversal muscles and longitudinal ventromedian muscles in the segments of the early segmentation stage but also in the esophagus area (Figure 3D,J,P). The *Uun_twist* gene was also expressed in the segmented region (Figure 3V,W). However, in the late segmentation stage, when the cilia were lost and the larvae transitioned to a benthic life, *Uun_st-mhc*, *Uun_troponin I*, and *Uun_calponin* were not expressed in the esophagus region, but strongly expressed in the segmentation region (Figure 3E,K,Q). Subsequently, in the worm-shaped larva, the *Uun_st-mhc*, *Uun_troponin I*, and *Uun_calponin* genes were expressed not only in the foregut muscles but also strongly in the body muscles (Figure 3F,L,R). In the case of the *Uun_twist* gene, no expression pattern was seen in the body muscles, but it was expressed between the muscle layer and internal organs (Figure 3X).

Previous studies reported that the body wall of *Urechis unicinctus* is composed of smooth somatic muscles [23], which are found in bilaterians with slow or sessile lifestyles [24,25,26,27]. Thus, to look for molecular evidence that the body wall of *U. unicinctus* consists of somatic muscles, we conducted double in situ hybridization (ISH) for *Uun_calponin* (smooth muscle maker) and *Uun_st-mhc* (striated muscle marker). The results showed that the expression of *Uun_calponin* and *Uun_st-mhc* genes was co-localized both at the segmented region and in the body wall muscles, including the foregut, in the late-segmentation larva and the worm-shaped larva, respectively (Figure 3X,Z,A’,B’). Interestingly, *Uun_st-mhc* and *Uun_calponin* were co-expressed in the *U. unicinctus* body wall smooth muscles. In *Platynereis dumerilii* belonging to polychaete, *Pdu_st-mhc* and *Pdu_troponin I* were mainly expressed in somatic muscle and foregut musculature, and *Pdu_calponin* was mostly expressed in visceral muscles, midgut, and hindgut, showing the similar expression pattern of striated and smooth muscle genes in vertebrate [17]. In contrast, in *U. unicinctus*, *Uun_st-mhc* and *Uun_troponin I* were expressed in somatic musculature, transverse muscles, and ventromedian muscles. *Uun_calponin* gene was co-expressed in a region where the *Uun_stmhc* and *Uun_troponin I* genes were expressed, which is similar musculature as in flatworm [28,29,30] and ascidians [31,32] with slow and sessile lifestyles. In summary, our study indicates that the expression patterns of striated and smooth muscle genes in *U. unicinctus*, a species belonging to Annelida and related to polychaete, were different from those of *P. dumerilii*, which suggests that the musculature of *U. unicinctus* might be due to adaptations to lifestyle and environment. 

### 2.4. Composition of U. unicinctus Muscle Layer in Adults

**Figure 4:**
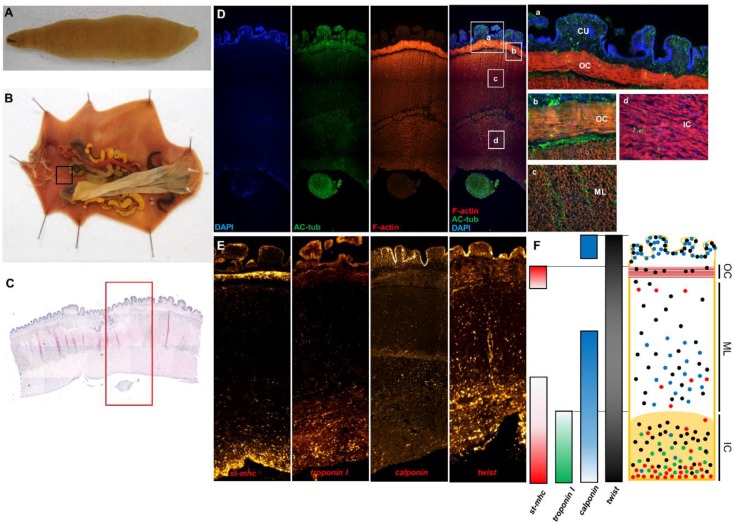
Morphology of an *U. unicinctus* adult and immunohistochemistry of an *U. unicinctus* adult muscle. (**A**) Morphology of *U. unicinctus* adult. (**B**) Anatomy of *U. unicinctus* adult. (**C**) H&E staining of muscle tissue (region is the black box in Figure 4B) with the ventral nerve cord. (**D**) Immunostaining and F-actin staining using anti-acetylated tubulin and phalloidin (region is the red box in Figure 4C). (**D**,**a**) Cuticle layer (CU) and outer circular muscle layer (OC) of *U. unicinctus*; (**D**,**b**) Outer circular muscle layer (OC); (**D**,**c**) Middle longitudinal muscle layer (ML); (**D**,**d**) Innermost circular muscle layer (IC). (**E**) Muscle-specific marker (*Uun_st-mhc*, *Uun_troponin I*, *Uun_calponin*, and *Uun_twist*) ISH in adult tissue. (**F**) Schematic summary of the expression patterns of muscle-specific genes in *U. unicinctus* adult tissue. (**C**–**E**) The images were taken with an EVOS FL Auto2 (Invitrogen) mosaic microscope.

To determine the general muscle composition in the body wall, we performed hematoxylin and eosin (H&E) staining and immunohistochemistry using anti-acetylated tubulin and phalloidin staining in adult muscle tissue (Figure 4C,D).

There were three layers of muscle in the body wall. The first layer (outer circular muscle layer; OC) consisted of many circular muscle fibers (Figure 4D,b), and in the cross-section, the second layer (middle longitudinal muscle layer; ML) presented cylindrical longitudinal muscles (Figure 4D,c). The third layer (innermost circular muscle layer; IC) showed circular muscles similar to the OC (Figure 4D,d). Overall, the muscular system in the body wall of *Urechis unicinctus* consisted of three muscle layers with tight muscle fibers and a single cuticle layer (CU), which was not stained by phalloidin. 

In addition, ISH was performed to confirm the expression pattern of muscle-specific marker genes (*Uun_st-mhc*, *Uun_calponin*, *Uun_twist*, and *Uun_troponin I*) in adult tissue (Figure 4E). The expression of the *Uun_twist* gene, a mesoderm marker, was evenly distributed throughout the muscle layers. In contrast, the smooth muscle marker *Uun_calponin* was broadly expressed in the CU, ML, and IC. *Uun_st-mhc* was strongly expressed in the OC and the innermost IC, whereas the *Uun-troponin I* gene was widely expressed only in the IC. These expression patterns indicate that *U. unicinctus* musculature is generally composed of smooth muscles [23]. However, it can be assumed that the muscles of *U. unicinctus* might be functionally divided according to muscle layers because the striated muscle and smooth muscle markers were co-expressed in the same region during development, whereas the striated and smooth marker genes were expressed in different muscle layers in adults. 

## 3. Materials and Methods

### 3.1. Sampling

Adult *Urechis unicinctus* species were collected from intertidal mud flats on the southern coast of South Korea and subjected to artificial insemination. Adult worms were dissected and the eggs and sperm were obtained from the gonads of each marine spoon worm and transferred to filtered seawater (FSW). Artificial fertilization was performed by mixing the sperm and eggs 1: 400 in FSW. *U. unicinctus* embryos were reared in artificial seawater (18 °C, pH 8, and salinity 30) in a plastic case. The late trochophores, a typical larval stage containing the intestinal tract, were fed microalga *Isochrysis galbana*. The developmental process from the zygote to the blastula stage lasted 14–16 hours, and cleavage occurred once per hour at 18 °C. The reared embryo samples were collected at the following stages: 0 h (unfertilized egg); 0.5 h post-fertilization (fertilized egg); polar body cell; 2-cell, 4-cell, 8-cell, 16-cell, and 32-cell stages; blastula and ciliated stages; early (day 1), middle (day 2), and late (day 5) trochophore stages; segmentation (day 30~45) and worm-shaped larval (day 60) stages.

### 3.2. Immunohistochemistry and Confocal Laser Scanning Microscopy (CLSM)

The specimens were anesthetized with isotonic 0.37 M MgCl_2_ for 10 min prior to fixation in 4% paraformaldehyde in PBT (phosphate-buffered saline +0.1% Tween-20) for 2 h at room temperature, followed by several rinses in PBT. 

After several washes in PBT, the animals were preincubated for 30 min at room temperature in PBT and then incubated overnight at 4 °C in primary antibodies (mouse anti-acetylated tubulin, Sigma-Aldrich, T7451; mouse anti-beta-tubulin, Sigma-Aldrich, T0198; or rabbit anti-serotonin, Sigma-Aldrich, S5545, Saint Louis, MO, USA) dissolved 1:500 in PBT. The animals were then rinsed several times in PBT, preincubated for 30 min at room temperature in PBT, and incubated overnight at 4 °C in secondary antibodies (goat anti-mouse conjugated with AlexaFluor488, ab150113, Abcam, Cambridge, UK; or AlexFluor568, A11011, Invitrogen, Waltham, MA, USA) dissolved in 1:1000 PBT. Eventually, the animals were rinsed several times in PBT, stained for cell nuclei with DAPI (1:1000 solution in PBS for 10 min), and mounted in 80% glycerol. 

The specimens were investigated at the cleavage stage (beta-tubulin antibody with DAPI), trochophore stage, segmentation larval stage (acetylated-tubulin, Sigma-Aldrich, Saint Louis, MO, USA with DAPI Sigma-Aldrich, Saint Louis, MO, USA and Texas Red™-X phalloidin staining, Invitrogen, Waltham, MA, USA), and worm-shaped larval stage (acetylated tubulin and serotonin antibody with DAPI and Texas Red™-X phalloidin staining).

### 3.3. Gene Identification, Gene Cloning, and Probe Synthesis

We isolated total RNA from *Urechis unicinctus* embryos of different developmental stages using TRIzol (Ambion, Austin, TX, USA). We selected mRNA from RNA using Oligo (dT) primers (Promega, Madison, WI, USA) and then conducted reverse transcription into cDNA (SuperScript II First-Strand Synthesis System for RT-PCR, Invitrogen, Waltham, MA, USA). The investigated *U. unicinctus* genes were isolated using gene-specific primers (*Uun-stmhc* forward: 5’- TCTGACGCTGAGGATCGCATT -‘3; *Uun-stmhc* reverse: 5’- GACTTCGGCACGCATAGCATT -‘3; *Uun-troponin I* forward: 5’- GAGAGAAGCCAGTTGGAAAGA -‘3; *Uun-troponin I* reverse: 5’- GATTTGAGCTGGTCTCTGAAC -‘3; *Uun-calponin* forward: 5’- TCAAGATGAGTCGTGCTGAGA-‘3; *Uun-calponin* reverse: 5’- ACCAATGACACCTTGACCCTC -‘3; *Uun-twist* forward: 5’- GATTACCCGGACACAGTCGAT -‘3; *Uun-twist* reverse: 5’- CCAATCAGTCCACTCAGCACC -‘3;) based on a sequenced RNA database available in our Laboratory of Cellular and Developmental Biology (LCDB, Cheongju, South Korea). The amplified genes fragments were subcloned into pGEM T vectors (Promega, Madison, WI, USA) and sequenced to confirm their identities. Then, these subcloned inserts were amplified using universal SP6 and T7 promoter primers (SP6 primer: 5’- TATTTAGGTGACACTATAG -3’; T7 primer: 5’- TAATACGACTCACTATAGG -3’). The PCR-amplified linear templates were used to synthesize an in vitro-transcribed antisense riboprobe (MEGAscript Transcription kit, Ambion, Austin, TX, USA). The genes are available under accession numbers 2271448 (*Uun-stmhc*), 2302567 (*Uun-troponin I*), 2271460 (*Uun-calponin*), and 2271494 (*Uun-twist*). Digoxigenin-labeled RNA probes were synthesized from the cloned fragments.

### 3.4. ISH in U. unicinctus

Fixation of the embryos from the zygote to the trochophore stage was performed with 4% paraformaldehyde (PFA, Sigma-Aldrich, Saint Louis, MO, USA) for 2 h. The ISH protocol for metameric and juvenile embryos was the same as mentioned above except that it was preceded by the collection and relaxation of embryos for 10 min in a relaxant solution (0.37 M MgCl_2_ in PBT) and fixation in 4% paraformaldehyde (PFA) for 2h. The fixed embryos were washed 5 × 5 min in 0.1% PBT (phosphate-buffered saline and 0.1% Tween 20) and permeabilized by treatment with 0.5 mg/mL proteinase K (Biofact, Daejeon, South Korea) in PBT for 5 min at room temperature. Proteinase K treatment was followed by 2 × 5 min rinses in a solution containing 2 mg/mL glycine in PBT, and 2 × 5 min washes in PBT at room temperature. Following additional 5 min washes in 0.5 mL 0.1 M triethanolamine buffer (TEA, pH 8.0, Sigma-Aldrich, Saint Louis, MO, USA), they were treated for 5 min with 0.5 mL of TEA supplemented with 3 μL of acetic anhydride. The embryos were then rinsed 3 × 5 min in PBT and post-fixed for 20 min in 4% PFA. The embryos were washed five times for 3 × 5 min each in PBT at room temperature and heated for 10 min at 80 °C in PBT to inactivate endogenous alkaline phosphatases. Further protocol conditions were identical to those described by Cho et al. [33], and double fluorescent in situ hybridization (FISH) was described by Kwak et al. [34].

### 3.5. Histological Analysis

To visualize the composition of the muscle structure, adult *Urechis unicinctus* were fixed in 4% PFA (Electron Microscopy Sciences, Hatfield, PA, USA) in 1X PBS overnight at 4 °C. For H&E staining and ISH, the muscles were dehydrated in a sucrose series and embedded in Optimal cutting temperature compound (VWR, Radnor, PA, USA) and rapidly frozen in liquid nitrogen. Cryosectioned samples (15-µm thick) were cut with a CM1520 cryostat (Leica, Wetzlar, HE, Germany) and stored at −70 °C until use.

## 4. Conclusions

Our study investigated the development of musculature from zygotes to adults in *Urechis unicinctus* using phalloidin staining, immunohistochemistry, and ISH. We observed the transversal muscles that formed the segments and the ventromedian muscles that formed the ventral nerve cord. After that, many muscles became cylindrical in shape. Muscle-related genes, such as *Uun_st-mhc*, *Uun_troponin I*, and *Uun_calponin*, showed co-localized expression patterns in the same regions. The musculature of *U. unicinctus* was similar to those of species with slow and sessile lifestyles. Interestingly, the muscle-related genes (*Uun_st-mhc*, *Uun_troponin I*, and *Uun_calponin*) were differentially expressed at each muscle layer of the adult body wall muscles, which is a different pattern from the common polychaete species in Annelida; thus, the musculature of *U. unicinctus* might be influenced by environment (Figure S1). This study will provide a basis for understanding the evolutionary changes in Echiura musculature.

## Supplementary Materials

The following are available online at https://www.mdpi.com/1422-0067/21/7/2306/s1, Figure S1: Phylogenetic trees of striated muscle (*Uun_st-mhc*, *Uun_troponin I*), smooth muscle (*Uun_calponin*) and mesoderm marker (*Uun_twist*).

## Abbreviations

c	cilia
at	apical tuft
glv	gastro-intestinal valve
np	nephridia
nt	neurotroch
pt	protroch
tt	telotroch
gl	ganglion
an	anus
bm	buccal muscle
bcm	buccal circular musculature
im	intestinal muscle
mc	myocyte
ptr	prototroch muscle ring
st	stomach
ls	larval stomach
int	intestine
tm	transverse musculature
vlm	ventrolateral longitudinal muscle
vmlm	ventromedian longitudinal muscle
vnc	ventral nerve cord
bp	blastpore
