# Supplementary material for: Muscular Development in Urechis unicinctus (Echiura, Annelida)
Source: Int J Mol Sci. 2020 Mar 26;21(7):2306. doi: 10.3390/ijms21072306 (PMC7178014; doi:10.3390/ijms21072306)
Supplement: Supplementary file 1 [file ijms-21-02306-s001.pdf]

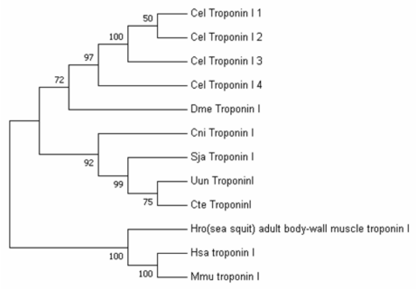

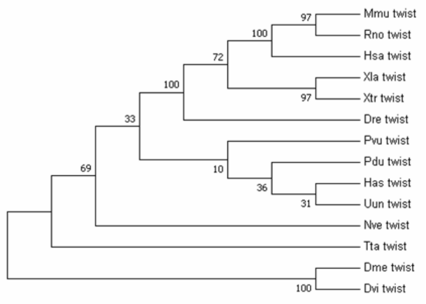

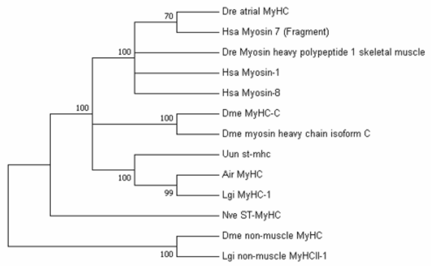

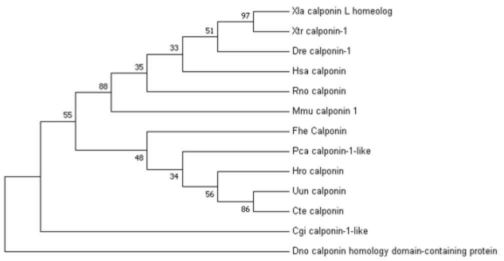

Supplementary data

For this purpose, all genes sequences were retrieved from the UniProt database and NCBI database. Alignment was carried out using the ClustalW software (http://clustalw.genome.jp). Phylogenetic analysis was performed by the Neighbor-joining method using the MEGA 7.0 program (http://www.megasoftware.net). Bootstrap analysis was performed with 1000 replicates Hsa: *Homo sapiens*; Dre: *Danio rerio*; Dme: *Drosophila melanogaster*; Cte: *Capitella teleta*; Pvu: *Patella vulgata*; Nve: *Nematostella vectensis*; Rno: *Rattus norvegicus* Pca: *Pomacea canaliculata*; Mmu: *Mus musculus*; Hro: *Helobdella robusta*; Cel: *Caenorhabditis elegans*; Air: *Argopecten irradians*; Cgi: *Crassostrea gigas*; Dno: *Drosophila novamexicana*; Fhe: *Fasciola hepatica*; Xtr: *Xenopus tropicalis* Xla: *Xenopus laevis*; Cni: *Chlamys nipponensis*; Sja: *Schistosoma japonium*; Hro(sea_squit): *Halocynthia roretzi*; Lgi: *lottia gigantea*; Pdu: *Platynereis dumerilii*; Tta: *Transennella tantilla*; Has: *Helix aspersa*; Dvi: *Drosophila virilis*.
